# QTL Mapping Using a High-Density Genetic Map to Identify Candidate Genes Associated With Metribuzin Tolerance in Hexaploid Wheat (*Triticum aestivum* L.)

**DOI:** 10.3389/fpls.2020.573439

**Published:** 2020-09-17

**Authors:** Ling Xu, Hui Liu, Andrzej Kilian, Roopali Bhoite, Guannan Liu, Ping Si, Jian Wang, Weijun Zhou, Guijun Yan

**Affiliations:** ^1^ Zhejiang Province Key Laboratory of Plant Secondary Metabolism and Regulation, College of Life Sciences and Medicine, Zhejiang Sci-Tech University, Hangzhou, China; ^2^ Faculty of Science, UWA School of Agriculture and Environment and The UWA Institute of Agriculture, The University of Western Australia, Crawley, WA, Australia; ^3^ Faculty of Science and Technology, Diversity Arrays Technology Pty Ltd., University of Canberra, Bruce, ACT, Australia; ^4^ Institute of Crop Science and Zhejiang Key Laboratory of Crop Germplasm, Zhejiang University, Hangzhou, China

**Keywords:** wheat, metribuzin tolerance, quantitative trait loci, marker validation, candidate genes

## Abstract

Tolerance to metribuzin, a broad-spectrum herbicide, is an important trait for weed control in wheat breeding. However, the genetics of metribuzin tolerance in relation to the underlying quantitative trait loci (QTL) and genes is limited. This study developed F_8_ recombinant inbred lines (RILs) from a cross between a highly resistant genotype (Chuan Mai 25) and highly susceptible genotype (Ritchie), which were used for QTL mapping of metribuzin tolerance. Genotyping was done using a diversity arrays technology sequencing (DArTseq) platform, and phenotyping was done in controlled environments. Herbicide tolerance was measured using three traits, visual score (VS), reduction of chlorophyll content (RCC), and mean value of chlorophyll content for metribuzin-treated plants (MCC). A high-density genetic linkage map was constructed using 2,129 DArTseq markers. Inclusive composite interval mapping (ICIM) identified seven QTL, one each on chromosomes 2A, 2D, 3A, 3B, 4A, 5A, and 6A. Three major QTL—*Qrcc.uwa.2AS*, *Qrcc.uwa.5AL*, and *Qrcc.uwa.6AL—*explained 11.39%, 11.06%, and 11.45% of the phenotypic variation, respectively. The 5A QTL was further validated using kompetitive allele-specific PCR (KASP) assays in an F_3_ validation population developed from Chuan Mai 25 × Dagger. Blasting the single-nucleotide polymorphisms (SNPs) flanking the QTL in the wheat reference genome RefV1.0 revealed SNP markers within or very close to annotated genes which could be candidate genes responsible for metribuzin tolerance. Most of the candidate genes were related to metabolic detoxification, especially those of P450 pathway and xenobiotic transmembrane transporter activity, which are reportedly key molecules responsible for herbicide tolerance. This study is the first to use specially developed populations to conduct QTL mapping on the metribuzin tolerance trait. The three major QTL and candidate genes identified in this study could facilitate marker-assisted metribuzin breeding in wheat. The QTL could be fine-mapped to locate the genes responsible for metribuzin tolerance, which could be introgressed into elite wheat cultivars.

## Introduction

Wheat (*Triticum aestivum* L.) is a major food crop worldwide, providing approximately 20% of the global daily consumption of calories and proteins (Monneveux et al., 2012; Shiferaw et al., 2013). Weeds are among the many restrictive factors that affect wheat yield, as they compete with the crop for light, water, nutrients, and space (Song et al., 2005). Weeds are generally controlled by herbicide application (Pan et al., 2012). Breeding wheat cultivars with high tolerance to herbicides will improve weed control efficiency and hence crop production.

Metribuzin is an excellent herbicide for controlling a wide range of weeds in dryland farming systems (Kleemann and Gill, 2008; Si et al., 2009). However, most wheat cultivars lack tolerance to this broad-spectrum herbicide. Our previous study identified cultivar Chuan Mai 25 with superior metribuzin-tolerance using a pre-emergence selection method (Bhoite et al., 2018); this cultivar is a valuable resource for increasing wheat tolerance to metribuzin. Genetic studies on metribuzin tolerance are limited. Bhoite et al. (2018) conducted a quantitative trait loci (QTL) mapping study using the International Triticeae Mapping Initiative (ITMI) population (Synthetic W7984 × Opata 85). Pilcher et al. (2017) identified 169 (upregulated) and 127 (downregulated) genes in a metribuzin-tolerant and a susceptible wheat genotype that had significant differential expression in response to metribuzin. Bhoite et al. (2019) further reported that the inheritance of pre-emergent metribuzin tolerance acted as an additive-dominance model in wheat and proposed putative genes through 90k SNP array analysis of seven cultivars with contrasting responses to metribuzin. However, no studies have constructed a genetic linkage map for metribuzin tolerance in wheat using a specially developed mapping population.

The genetic mechanism of metribuzin tolerance in wheat is not fully understood. Different mechanisms for metribuzin tolerance in weeds and crops have been reported. Metribuzin tolerance in weeds is mostly target site-based, targeting the chloroplast *psb*A gene encoding D1 protein, which inhibits photosynthesis at photosystem II (PSII) (Pan et al., 2012). Specific point mutation in the *psb*A gene conferring triazine resistance is mainly reported in weeds (Powles and Yu, 2010), while only a few mutations have been found in crops, such as triazine-tolerant canola (Reith and Straus, 1987). Most of the herbicide tolerance in crops is based on non-target site mechanisms. Villarroya et al. (2000) revealed that the inheritance of metribuzin tolerance in durum wheat involves many QTL in the nucleus, but not in the cytoplasm. Kilen and Barrentine (1983) studied the inheritance of metribuzin tolerance in wild soybean and reported that the trait is controlled by alleles at the same locus as the *Hm* gene. The mechanism of metribuzin tolerance in narrow-leafed lupin is linked to a non-target site, and likely to be based on P450-mediated metribuzin metabolism (Pan et al., 2012). Moreover, Javid et al. (2017) identified major QTL and the underlying cytochrome P450 genes associated with metribuzin tolerance in field pea, suggesting a non-target site mechanism in the crop.

Diversity arrays technology sequencing (DArTseq) is a high-throughput genotyping method used extensively for mapping, genetic diversity, and association mapping studies (Jaccoud et al., 2001; Marone et al., 2012). Gawroński et al. (2016) reported that DArT markers effectively target gene space in the large, complex, and repetitive rye (*Secale cereale* L.) genome. Xia et al. (2005) revealed DArT as effective markers for high-throughput genotyping of cassava (*Manihot esculenta*) and its wild relatives. Marone et al. (2012) demonstrated that wheat DArT markers represent the main features of the genome and could serve as an ideal tool for identifying candidate genes. The sequence knowledge of DArT markers can provide functional meaning to those markers facilitating candidate gene identification (Marone et al., 2012; Aitken et al., 2014).

This study aimed to 1) use the specially developed F_8_ RIL populations to conduct QTL mapping on metribuzin tolerance; 2) develop a high-density genetic linkage map of hexaploid wheat with DArTseq markers; 3) identify QTL for metribuzin tolerance traits, including visual score (VS), reduction of chlorophyll content (RCC), and mean value of chlorophyll content for metribuzin-treated plants [mean chlorophyll content (MCC)]; and 4) identify candidate genes associated with the QTL for metribuzin tolerance.

## Materials and Methods

### Plant Material

Seeds of four cultivars of *Triticum aestivum* L.—Chuan Mai 25, Ritchie, Dagger, and Spear—were obtained from the Australian Winter Cereals Collection. The responses of the four cultivars to metribuzin have been reported or screened among 946 genotypes, as described by Bhoite et al. (2017). Chuan Mai 25 is among the most tolerant to metribuzin, whereas Ritchie, Dagger, and Spear are susceptible to metribuzin. The four cultivars were selected to develop one mapping population (Chuan Mai 25 × Ritchie) and two validation populations (Chuan Mai 25 × Dagger and Chuan Mai 25 × Spear).

### Population Development

One hundred and thirty-seven F_8_ recombinant inbred lines (RILs), developed from a cross between the metribuzin tolerant cultivar Chuan Mai 25 and susceptible cultivar Ritchie (Bhoite et al., 2017), were used for QTL mapping of metribuzin tolerance. Following the QTL mapping, crosses were made between Chuan Mai 25 and two susceptible genotypes (Spear and Dagger) to develop populations to validate the phenotypic effects of the identified QTL in different genetic backgrounds. Seventy lines were randomly selected from each F_3_ validation population for the validation experiment.

### Plant Growth and Treatment

The F_8_ RILs were grown in seedling trays with 5 × 6 cells (50 × 50 × 60 mm) filled with homogenous sand. One seed per cell was sown for each of the RILs in a greenhouse at The University of Western Australia (31°57′S, 115°47′E) where they were watered every 2 days. Three biological replicates (in different trays) with six plants in a row (within a tray) for each replicate were used for the control and treatment, respectively. The trays were sprayed with water to 100% field capacity one day before the herbicide application. The pre-emergent treatment of metribuzin (400 g ai ha^−1^) was as described in Bhoite et al. (2018). Specifically, metribuzin was sprayed in two passes, with the cabinet sprayer calibrated to deliver a spray volume of 106.19 L ha^−1^, 50 cm above the target, with a forward speed of 3.6 km h^−1^ and a pressure of 200 kPa in two flat-fan nozzles (TeeJet XR11001 flat-fan, Spraying Systems Co, Wheaton, IL, USA) (**Figure S1**, doi: 10.6084/m9.figshare.12570113). After being sprayed, the seedlings were transferred to a phytotron set at 25/15°C (day/night), 14-h/10-h photoperiod, and 800 µmol m^−2^ s^−1^ intensity. The plants were watered every two days. About 15 days after treatment, samples were selected for the phenotypic evaluation. The same spray and glasshouse settings were used to evaluate the effects of metribuzin in the two validation populations.

### Phenotyping

The chlorophyll content of the RILs was determined using a portable Minolta SPAD-502 chlorophyll meter (Spectrum Technologies, Inc., Plainfield, IL, U.S.) to evaluate the phytotoxic effects of metribuzin on wheat seedlings, with three replicate reads for each measurement. The average read from a fully unfolded leaf represented the final result, which is linearly correlated with plant chlorophyll concentration (Hamblin et al., 2014; Bhoite et al., 2018). The effects of metribuzin were assessed using three parameters: RCC, relative to the control (high RCC values representing greater susceptibility and low RCC values representing greater tolerance); MCC, for metribuzin-treated plants; and VS from 0 (100% senescence/phytotoxicity) to 10 (no senescence/no sign of phytotoxicity) to estimate leaf senescence (for further details, see Bhoite et al. (2018). The control plants remained healthy, with a VS of 10 for all plants.

### Phenotypic Data Analysis

A phenotypic data analysis was conducted using GenStat statistical software 17^th^ edition (VSN International, 2014). Analysis of variance (ANOVA) was performed based on the fixed effects model of Y_ij_ = μ + g_j_ + Ɛ_ij_, where Y_ij_ is the observed phenotypic mean, μ is the population mean, g_j_ is the effect due to the j^th^ genotype, and Ɛ_ij_ is the random error. Heritability was measured using the formula of ${\text{h}}^{2}=\delta_{g}^{2}/(\delta_{g}^{2}+\delta_{e}^{2})$, where $\delta_{g}^{2}$ and $\delta_{e}^{2}$ are estimated genotypic and error variances as $\delta_{g}^{2}=\frac{MSg-MSe}{r}$ and $\delta_{e}^{2}=\frac{MSe}{r}$, respectively, where *MSg* is the mean square of the RILs, *MSe* is the residual error, and r is the number of replicates (Nyquist and Baker, 1991; Onyemaobi et al., 2018).

### Genotyping and QTL Mapping

Genomic DNA was extracted from the leaves of 15-day-old seedlings of individual plants from parental lines of Chuan Mai 25, Ritchie, Dagger, and Spear, and each of the 137 F_8_ mapping population and 140 F_3_ validation populations (70 lines from Chuan Mai 25 × Dagger and 70 lines from Chuan Mai 25 × Spear) using the cetyl trimethyl ammonium bromide (CTAB) method with some modifications.

Total genomic DNA quality and quantity were checked on NanoDrop™ 2000 Spectrophotometer (Thermo Fisher Scientific Australia). DArT sequencing was conducted by Diversity Arrays Technology Pty Ltd. (Canberra, Australia) following the protocol described on the company’s website (https://www.diversityarrays.com/). The SNPs within the available genomic fragments were used for the linkage map construction. The DArT sequencing generated 28,668 polymorphic SNP markers. The raw data were sorted according to the marker alleles for each RIL, which were compared to that of their parental lines, Chuan Mai 25 and Ritchie. Those with the Chuan Mai 25 allele were coded as A, Ritchie as B, and heterozygous loci were coded as H. The data were then inputted into IciMapping 4.0 software, with redundant markers deleted using the “bin” function, based on the anchor information to generate the map file. After performing grouping, ordering, and rippling, a linkage map was generated.

QTL analysis was conducted for the three trait values using the inclusive composite interval mapping-additive and dominance (ICIM-ADD) method of IciMapping 4.0. The logarithm of odds (LOD) > 2.5 was selected to declare significant QTL for the VS, RCC, and MCC traits. The R^2^ rate represents the percentage of variance explained by each QTL in proportion to the total phenotypic variance (Bearzoti and Vencovsky, 1998). The QTL were classified as major when their R^2^ value was more than 10%. The graphical representations of QTL on linkage groups were drawn using MapChart 2.32 software (Voorrips, 2002).

### QTL Validation

Kompetitive allele-specific PCR (KASP) markers developed from the flanking markers of the identified QTL were used to genotype the segregating lines in the validation populations. The KASP reagents were obtained from Geneworks Pty Ltd, Australia. In order to detect the markers, PCR was performed in a total volume of 10 μl, containing 15-ng genomic DNA as a template, 5-μl KASP 2 × reaction mix, 0.14 μl, primer mix, and DNase/RNase-free water. The PCR reactions were amplified in an Eppendorf Mastercycler programmed as 94°C for 15 min, 10 cycles of 94°C for 20 s and 61°C (with a decrement of −0.6°C per cycle until 55°C) for 60 s, and 26 cycles of 94°C for 20 s and 50°C for 60 s.

The KASP genotyping assays were read by an Applied Biosystems 7500 Fast Real-Time PCR System. Two F_3_ cross populations (Chuan Mai 25 × Dagger and Chuan Mai 25 × Spear) were genotyped and phenotyped to validate a major QTL on chromosome 5A, using marker DArT1101715. The mean phenotypic performances of the lines based on the two types of allele combinations (AA and aa) were compared using the student’s t-test (P < 0.05).

### Identification of Putative Candidate Genes

To identify putative coding gene regions, the flanking DArT marker sequences of the identified QTL were used to perform a BLASTN search (expect threshold = 10) against the wheat genome database Refv1.0 using the website blast tool (https://urgi.versailles.inra.fr/blast/?dbgroup=wheat_iwgsc_refseq_v1_chromosomes&program=blastn). Genes within the QTL interval with known functions for metabolic detoxification and stress resistance, especially those having SNP variation within the genes, were considered potential candidate genes for metribuzin tolerance. Gene IDs beginning with Traes were obtained from JBrowse. Stress-relevant genes were selected from the other two protein databases (https://www.uniprot.org; http://www.ebi.ac.uk/interpro/entry/IPR026509) to obtain more information, including those with protein domain, family, molecular, and biological functions of the candidate genes.

## Results

### Phenotypic Variation of Wheat Under Metribuzin Stress

**Figure 1:**
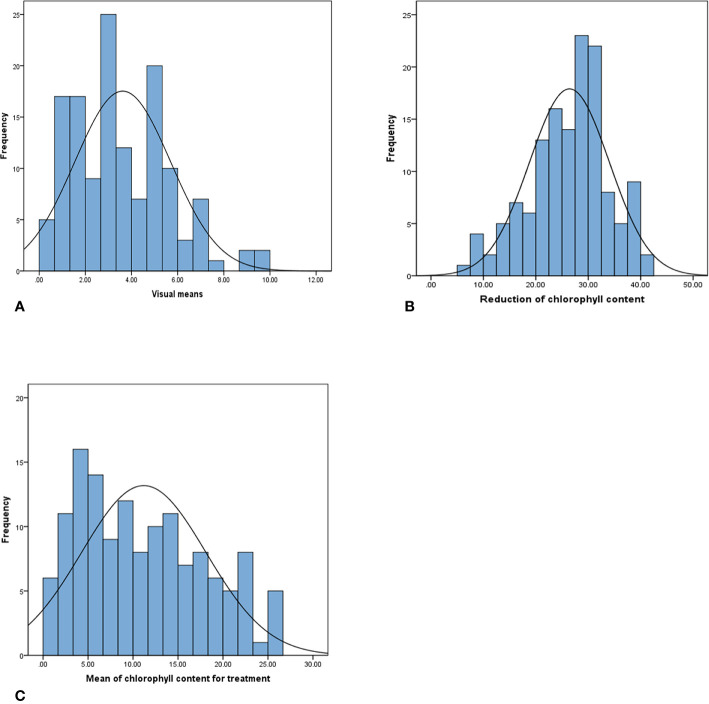
Phenotypic distribution of **(A)** visual score, **(B)** reduction of chlorophyll content, and **(C)** mean value of chlorophyll content for metribuzin-treated plants in 137 RILs of the Chuan Mai 25 × Ritchie mapping population.

The phenotypic data analysis revealed a normal frequency distribution among the 137 F_8_ RILs of Chuan Mai 25 × Ritchie for the metribuzin response (**Figure 1**). A wide range of phenotypic variation was observed in the RILs for the three traits in the control and metribuzin treatment (**Table S1**, doi: 10.6084/m9.figshare.12570113). After being treated with metribuzin, the MCC, RCC, and VS values among the RILs ranged from 0.43–26.33, 5.70–41.47, and 0.00–10.00, respectively. Broad sense heritability for chlorophyll content was 83% in the metribuzin treatment and 60% under normal growing conditions, indicating genetic factors strongly influence chlorophyll content variation in the mapping population (**Table 1**).

**Table 1:** Analysis of variance for metribuzin tolerance and associated traits and their heritability estimates in the 137 RILs of Chuan Mai 25 × Ritchie mapping population.

Category	*MSg*	*MSe*	$\delta_{g}^{2}$	$\delta_{e}^{2}$	$\delta_{p}^{2}$	h^2^
Visual score	12.96**	2.73	3.41	0.91	4.32	0.79
Mean value of chlorophyll content for treatment	143.33**	24.75	39.53	8.25	47.78	0.83
Mean value of chlorophyll content for control	45.09**	18.16	8.98	6.05	15.03	0.60
Reduction of chlorophyll content	174.75**	45.19	43.19	15.06	58.25	0.74

MSg, mean square of genotype; MSe, mean square of random error; $\delta_{g}^{2}$, estimated genetic variance; $\delta_{p}^{2}$, estimated phenotypic variance; $\delta_{e}^{2}$, estimated error variance; h^2^, broad-sense heritability.

** indicates significant difference at p < 0.01.

### The Chuan Mai 25 × Ritchie Map

A total of 2,129 polymorphic SNP markers were used to generate the genetic linkage map, which were distributed over 21 linkage groups and assigned to the 21 wheat chromosomes based on the anchor DArTseq markers. The constructed genetic map covered 13,599 cM of the genome, with an average distance of 6.39 cM between adjacent markers. Linkage analysis assigned 38.99%, 36.92%, and 24.10% of the markers to the A, B, and D genomes, respectively. The DArTseq markers were not uniformly distributed on different chromosomes; chromosome 4D had the fewest markers (32), and chromosome 1A had the most markers (152). Moreover, chromosome 4D had the largest marker gap (15.85 cM), and chromosome 3A had the smallest gap (3.69 cM).

### QTL for Metribuzin Tolerance

**Figure 2:**
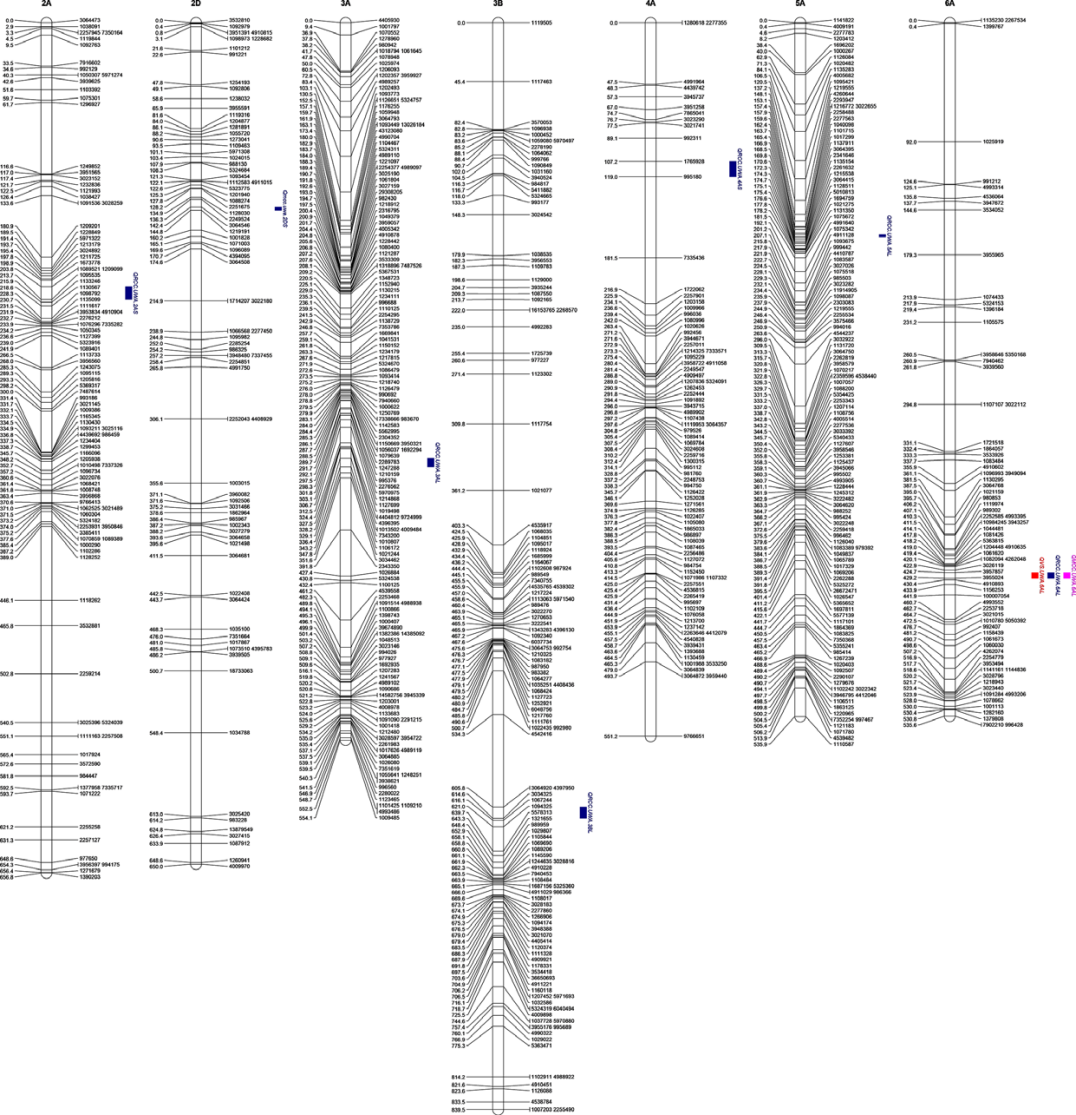
Locations of detected QTL for metribuzin tolerance in Chuan Mai 25 × Ritchie RILs for visual score (VS), reduction in chlorophyll content (RCC), and mean value of chlorophyll content for metribuzin-treated plants (MCC). QTL are indicated by solid bars; blue bars indicate QTL for RCC, red bars indicate QTL for VS and pink bars indicate QTL for MCC. Map distances are indicated on the left in Kosambi centimorgans and DArT markers are indicated on the right of each chromosome.

The QTL for metribuzin tolerance was mapped by integrating appropriate phenotypic and genotypic data (**Tables S2**, **S3**, doi: 10.6084/m9.figshare.12570113). In Chuan Mai 25 × Ritchie, seven QTL for metribuzin tolerance were detected for VS, RCC, and MCC, with one each on chromosomes 2A, 2D, 3A, 3B, 4A, 5A, and 6A (**Table 2** and **Figure 2**). The LOD scores ranged from 2.60–5.49, explaining 8.62–11.45% of the phenotypic variation in RCC (**Table 1**). The seven QTL were mostly located on long chromosome arms, apart from *QRCC.UWA.2AS*, *QMCC.UWA.2DS*, and *QRCC.UWA.4AS* that were located on the short chromosome arms of 2A, 2D, and 4A at 203.8 cM, 144.0 cM, and 107.3 cM, respectively. The QTL on the long chromosome arm of 6A (*QVS.UWA.6AL*, *QRCC.UWA.6AL*, and *QMCC.UWA.6AL*) were detected at 429 cM for all three traits (VS, RCC, and MCC), explaining up to 11.45% of the phenotypic variation, with a LOD score of 5.49. Another major QTL (*QRCC.UWA.5AL*) contributed 11.06% of the phenotypic variation, with a LOD score of 5.27, which was mapped on the long chromosome arm of 5A at 164 cM for RCC. The other two QTL (*QRCC.UWA.3AL* and *QRCC.UWA.3BL*) were detected on the chromosomes of 3AL and 3BL at 486.4 cM and 612 cM, respectively. Among the seven identified QTL for metribuzin tolerance, those on chromosomes 2AS, 2DS, 3BL, 4AS, 5AL, and 6AL gained favorable alleles from Chuan Mai 25, while that on 3AL gained its favorable allele from Ritchie (**Table 1**).

**Table 2:** Quantitative trait loci (QTL) for metribuzin tolerance identified in the Chuan Mai 25 × Ritchie mapping population.

Trait	Chromosome	QTL	QTL position (cM)	Left marker	Right marker	QTL interval (cM)	LOD	R^2^ (%)	Additive effect
VS	6A	**QVS.UWA.6AL**	429.0	3957857	3955024	426.50–430.50	3.05	10.83	0.66
RCC	2A	QRCC.UWA.2AS	203.8	1209099 or 1089521	1095535	203.75–213.66	3.63	11.39	–2.76
	3A	QRCC.UWA.3AL	486.4	2253468	1091514 or 4988938	482.34–489.79	3.91	9.06	2.15
	3B	QRCC.UWA.3BL	612.0	4397950	3034325	605.80–614.62	2.87	7.65	–2.36
	4A	QRCC.UWA.4AS	107.3	1765928	995180	107.24–118.99	2.60	8.62	–2.85
	5A	QRCC.UWA.5AL	164.0	1101715	1017299	163.71–165.44	5.27	11.06	–2.65
	6A	**QRCC.UWA.6AL**	429.0	3957857	3955024	424.73–429.20	5.49	11.45	–2.65
MCC	2D	QMCC.UWA.2DS	144.0	3064546	1219191	142.37–144.75	3.30	8.94	2.41
	6A	**QMCC.UWA.6AL**	429.0	3957857	3955024	424.73–429.20	3.22	8.37	1.93

VS, visual score; RCC, reduction of chlorophyll content; MCC, mean value of chlorophyll content for metribuzin-treated plants; LOD, logarithm of odds (LOD > 2.5 selected to declare significant QTL for the VS, RCC, and MCC traits); R^2^, percentage of variance explained by each QTL in proportion to the total phenotypic variance.

QTL in bold have the same marker interval.

### Validation of the Major QTL for Metribuzin Tolerance Under Herbicide Stress

A major QTL (*QRCC.UWA.5AL*) explained 11.06% of the phenotypic variation, with a high LOD score of 5.27 detected for RCC, and was contributed by the resistant genotype Chuan Mai 25. This QTL was selected for validation in 70 randomly selected lines from the two F_3_ populations (Chuan Mai 25 × Dagger and Chuan Mai 25 × Spear). DArT1101715, located just 0.29 cM from the peak QTL location, was used to validate the 5A QTL.

**Figure 3:**
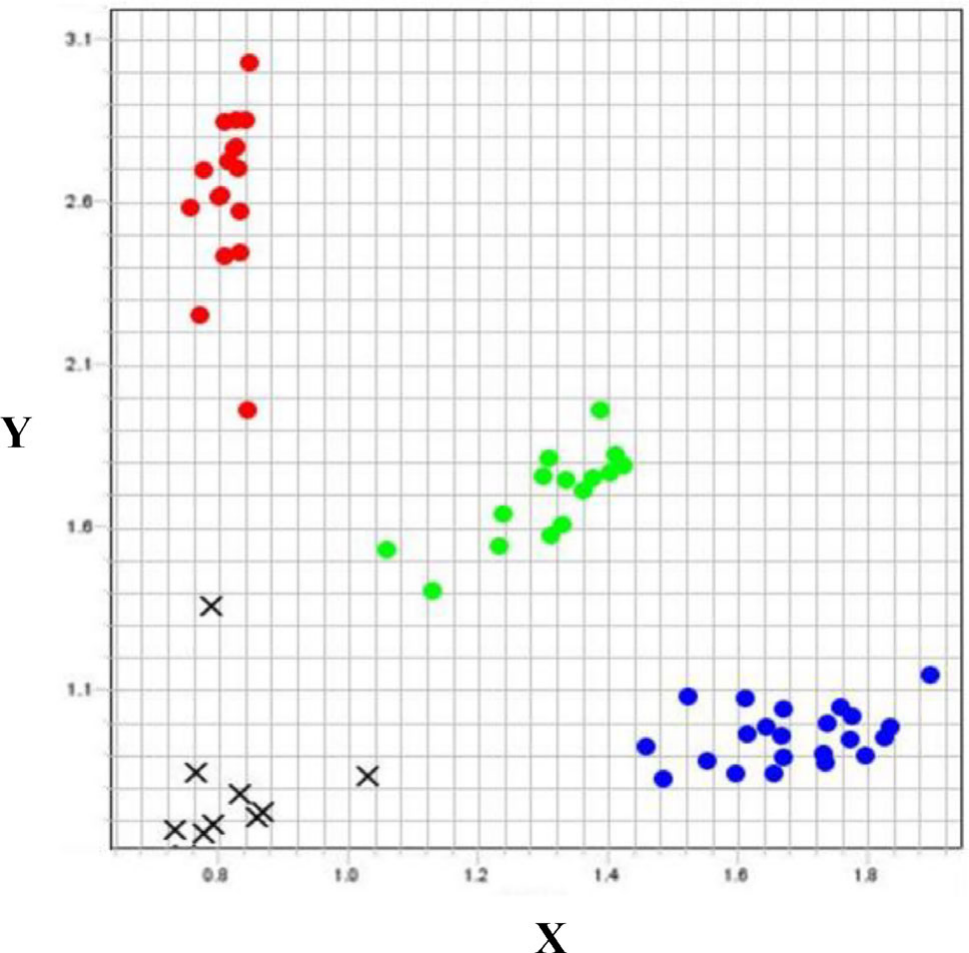
Genotyping plot of KASP assay for DArT1101715 on Chuan Mai 25 × Dagger. X-axis: Allele1, reported by FAM fluorescence; Y-axis, Allele 2, reported by HEX fluorescence; Red dots, homozygous allele group 1 (susceptible); Blue dots: homozygous allele group 2 (resistant); Green dots, heterozygous alleles; Black crosses: no call.

The validation lines with different alleles for each DArT marker were separated into allele group 1 (susceptible allele) and allele group 2 (resistant allele). DArT1101715, targeting *QRCC.UWA.5AL* (**Figure 3**), had a 21.2% higher average VS and 27.1% higher chlorophyll content for the Chuan Mai 25 × Dagger progenies with homozygous alleles from Chuan Mai 25 (allele group 2) than Ritchie (allele group 1). However, no polymorphism occurred for the marker in the Chuan Mai 25 × Spear population. As DArT1101715 is 0.29 cM from the QTL peak, monomorphism of this marker did not necessarily mean no variance at the *QRCC.UWA.5AL* locus, suggesting that more markers need to be developed for this locus.

### Putative Candidate Genes

From the seven QTL for metribuzin tolerance in Chuan Mai 25 × Ritchie, the flanking markers of each QTL were blasted in the URGI-JBrowse and Uniprot databases, which identified several putative candidate genes related to metabolic detoxification, including cytochrome P450 pathway, xenobiotic transmembrane transporter, and defense responses (**Table 3**).

**Table 3:** Putative candidate genes for the identified QTL conferring metribuzin tolerance in wheat.

QTL name	Refv1.0 gene ID	Gene physical position	Gene function (resources)	Gene length (bp) and direction	UniProtKB gene ID	Subcellular location	Molecular function	Biological process
*Qrcc.uwa.2AS* and *Qmcc.uwa.2AS*	TraesCS2A01G367800	611347900–611356150; 8,144 bp away from DArT1095535	Cytochrome P450 (Refv1.0; InterPro; Pfam)	8,258+	A0A3B6B237_WHEAT	Other locations: integral component of membrane	Uncharacterized protein; iron ion binding; monooxygenase activity	
*Qrcc.uwa.3AL*	TraesCS3A01G329100	574327750–574342550; 3,715 bp away from DArT2253468	Negative regulation of translation (GO:0017148), rRNA N-glycosylase activity (GO:0030598)	14,770+	A0A3B6EK35_WHEAT	Other locations	Catalytic activity; toxin activity	Defense response; negative regulation of translation
	TraesCS3A01G300400	534467440–534469620; overlap with DArT1091514	Antiporter activity; xenobiotic transmembrane transporter activity (InterPro)	2,157+	A0A3B6EJ09_WHEAT	Other locations: integral component of membrane	Protein detoxification (multidrug and toxic compound extrusion protein)	
	TraesCS3A01G289400	517996360–517997250; 1,075 bp away from DArT4988938	Transcriptional repressor – ovate; (Refv1.0; InterPro)	891+	A0A3B6EKS8_WHEAT		Negative regulation of transcription	
*Qrcc.uwa.3BL*	TraesCS3B01G303100	487203293–487224910; overlap with DArT3034325	Ion transport protein; ion channel activity (Refv1.0; InterPro)	21,618+	A0A077RUK6_WHEAT	Golgi apparatus; vacuole; other locations: integral component of membrane	Uncharacterized protein; calcium ion binding; identical protein binding; ion channel activity	Ion transport; calcium-mediated signaling; regulation of jasmonic acid biosynthetic process
*Qrcc.uwa.4AS*	TraesCS4A01G452300	717296015–717297935; overlap with DArT1765928	F-box-like domain superfamily (IPR036047); Transmembrane protein 183 (IPR026509) (InterPro)	1,920+	A0A3B6I2A1_WHEAT		Uncharacterized protein	
	TraesCS4A01G007400	4775750–4779350; 1,626 bp away from DArT995180	Polypeptide_domain (Refv1.0; InterPro; Pfam)	3,590–	A0A3B6HML7_WHEAT	Other locations: integral component of membrane	3-ketoacyl-CoA synthase; transferase activity	Fatty acid biosynthesis
*Qrcc.uwa.5AL*	TraesCS5A01G377600	575323152–575328643; 1677 bp away from DArT1101715	Auxin canalization, plant pleckstrin homology-like region (Refv1.0)	5,492+	A0A3B6KPS3_WHEAT		Uncharacterized protein	
*Qvs.uwa.6AL*, *Qrcc.uwa.6AL*, and *Qmcc.uwa.6AL*	TraesCS6A01G222800	416654584–416658623; overlap with DArT3957857	Aromatic amino acid lyase (Refv1.0; InterPro; Pfam)	4,040+	A0A3B6NRY8_WHEAT	Cytoplasm	**Phenylalanine ammonia-lyase,** catalytic activity	Trans-cinnamate biosynthesis

+/− represent direction (forward/reverse) on the strand; bp, base pairs.

For *Qrcc.uwa.2AS* and *Qmcc.uwa.2AS*, the blastN search of the markers in Refv1.0 identified three potential candidate genes with known functions in stress tolerance, including a gene coding cytochrome P450 which regulates oxidoreductase activity. *Qrcc.uwa.3AL* was also co-located with three candidate genes related to the defense response, negative regulation of translation, and protein detoxification. One candidate gene was identified from *Qrcc.uwa.3BL*, which is involved in ion transport, regulation of jasmonic acid (JA) biosynthetic, and calcium-mediated signaling. Two important candidate genes were identified from *Qrcc.uwa.4AS* with known functions of F-box-like domain superfamily, transmembrane protein, and fatty acid biosynthesis. One candidate gene was identified from *Qrcc.uwa.5AL*, which codes auxin canalization and plant pleckstrin homology-like region, and plays an important role in plant growth and resistance. One potential gene was identified from *Qvs.uwa.6AL, Qrcc.uwa.6AL*, and *Qmcc.uwa.6AL*, which was located in the cytoplasm with a phenylalanine ammonia-lyase function involved in trans-cinnamate biosynthesis.

## Discussion

This study is the first to use specially developed population lines to conduct QTL mapping on metribuzin tolerance in wheat. Seven QTL for metribuzin tolerance were identified from the F_8_ RILs derived from a highly metribuzin-resistant genotype (Chuan Mai 25) and a highly susceptible genotype (Ritchie). The major 5A QTL was further validated in an F_3_ validation population, developed from Chuan Mai 25 × Dagger crosses, using KASP assays. Most of the identified QTL were related to metabolic detoxification, especially the P450 pathway and xenobiotic transmembrane transporter activity.

RCC offers a rapid and non-destructive method for evaluating senescence in wheat seedlings in the metribuzin treatment as chlorophyll content and senescence are highly correlated (Bhoite et al., 2018). Metribuzin mainly inhibits photosynthesis by competing with plastoquinone at the plastoquinone-binding site on the D1 protein in the PSII complex (Pan et al., 2012). A series of reactions occur in plants in response to herbicides, which reduce chlorophyll content due to the production of active oxygen species (Xu et al., 2015). Higher RCC values in this study indicated that part of the PSII system was damaged, as reported under various stresses in plants (Gill et al., 2015; Xu et al., 2015; Wang et al., 2016).

One major QTL was located on the short arm of wheat chromosome 2A. This genomic region harbored cytochrome P450 genes, which are widely reported as responsible for tolerance to different stresses, including herbicides. Cytochrome P450 enzymes can accelerate metabolism and facilitate the detoxification of certain herbicide molecules (Zimmerlin and Durst, 1992). Bhoite et al. (2019) also reported that the candidate gene on chromosome 2A has the important biological function of metabolic detoxification, which is consistent with our findings. An increase in P450-dependent metabolism was first reported in *Lolium rigidum* herbicide-resistant biotypes from Australia and *Alopecurus myosuroides* biotypes from Europe (Holt et al., 1993). Siminszky (2006) reported plant cytochrome P450-mediated herbicide metabolism and the pivotal role of plant P450s in herbicide metabolism. Furthermore, enhanced detoxification in herbicide-resistant weeds was associated with elevated levels of P450 activity (Siminszky, 2006). Cytochrome P450 enzymes facilitate herbicide metabolism in many crops, including wheat, maize, and sorghum (Werck-Reichhart et al., 2000). Pan et al. (2012) indicated that the mechanism of metribuzin tolerance in lupin is attributed to P450-mediated metabolism. Yu and Powles (2014) revealed that herbicide resistance is often due to metabolic resistance and that cytochrome P450 monooxygenase is usually implicated in herbicide metabolic resistance. Future studies on metabolic pathways could identify the specific genes related to herbicide metabolic resistance (Fang et al., 2018; Yang et al., 2018a; Yang et al., 2018b).

A major QTL (*Qrcc.uwa.3AL*) contributing to variations in metribuzin tolerance was located on wheat chromosome 3A, a region known to carry major genes related to tolerance and adaptability (Kulwal et al., 2005). The candidate gene encodes a ribosome-inactivating protein related to the defense response. The results are consistent with those of Yang et al. (2016), who reported a positive correlation between the level of ribosome-inactivating protein (RIP) and resistance to cucumber mosaic virus in *Momordica charantia*. RIPs can induce apoptosis in a wide variety of cells (Narayanan et al., 2005), which plays a role in the regulation of cell death induced by the herbicide. *Qrcc.uwa.3AL* was also related to antiporter activity and xenobiotic transmembrane transporter activity to realize the protein detoxification by multidrug and toxic compound extrusion (MATE) protein. Transporter proteins from the MATE family are vital for metabolite transport in plants (He et al., 2010).

The genomic region of *Qrcc.uwa.3BL* harbors genes encoding ion transport proteins which operate in the ion channel activity and are related to JA regulation. JA is a plant hormone synthesized in the chloroplast membrane, which plays an important role in the abiotic stress responses (Kazan, 2015). It is also involved in a series of stress-related processes, such as stomatal closure and the detoxification activity of antioxidant enzymes, which could increase plant tolerance to various abiotic stresses (Zhou et al., 2015; Ahmad et al., 2016). Hu et al. (2019) indicated that *SlF3HL* is a positive regulator of chilling stress tolerance in tomato, possibly by regulating JA biosynthesis and signaling. Chapman et al. (2018) revealed that JA contributed to soybean’s tolerance to soybean aphid. Interestingly, JA triggered stress tolerance to the herbicide imazapic in tobacco (Kaya and Doganlar, 2016). JA enhanced plant tolerance to stresses in wheat by increasing antioxidant activity (Qiu et al., 2014). Thus, the 3BL QTL related to ion transport and JA pathway might play a vital role in metribuzin tolerance in wheat.

For the 4AS QTL, the candidate gene *TraesCS4A01G452300* belongs to the F-box-like domain superfamily. Gupta et al. (2015) indicated that F-box genes played a crucial role in the response to biotic and abiotic stresses in chickpea. The wheat F-box protein gene *TaFBA1* was related to plant tolerance to heat stress (Li et al., 2018). Various abiotic stresses induced the expression of *TaFBA1*, including drought, NaCl, and abscisic acid (ABA) (Zhou et al., 2014). Peng et al. (2014) demonstrated that F-box proteins conferred salinity tolerance in *Arabidopsis*. Moreover, the F-box protein COI1 plays an vital role in the jasmonate-signaling pathway and regulates primary carbohydrate metabolism in tobacco (Wang et al., 2014). F-box proteins also enhance polyphenol production and UV tolerance in *Arabidopsis* (Zhang et al., 2015). Another candidate gene *TraesCS4A01G007400* in the QTL region coding the important 3-ketoacyl-CoA synthase (KCS) in the fatty acid biosynthesis. KCS is a key enzyme for the synthesis of long-chain fatty acids and wax, which serve as the first line of defense against pathogens, phytophagous insects, and environmental stresses, such as drought, UV damage, and frost (Todd et al., 1999; Joubès et al., 2008). Todd et al. (1999) demonstrated that *KCS1* plays a vital role in the resistance of *Arabidopsis thaliana* to low humidity stress at a young age. Azachi et al. (2002) cloned a *KCS* gene from *Dunaliella salina*, which has outstanding salt tolerance, that might play a role in balancing adverse external osmotic pressure. Fan et al. (2018) found that stress conditions dramatically increased the expression of the *MaKCS* gene in *Mychonastes afer*.

The genomic region of major QTL *Qrcc.uwa.5AL* harbored genes coding auxin canalization and related to the plant pleckstrin homology-like region. Lee et al. (2014) demonstrated the canalization-based vein formation in a growing leaf and cell division along the shortest axis contributed to the relaxation of stress, as reported by Alim et al. (2012). Auxin acts as a general coordinator of plant growth and development (Leyser, 2018). Therefore, auxin canalization may be crucial for herbicide transportation and cell metabolic detoxification.

The candidate gene for the major 6AL QTL was related to coding aromatic amino acid lyase involved in trans-cinnamate biosynthesis. The aromatic amino acid has been related to the detoxification of glyphosate (Samsel and Seneff, 2013), and regulation of salt stress in wheat (Jiang et al., 2017). One flanking DArT marker of the QTL overlapped the mRNA CJ661337 (https://www.ncbi.nlm.nih.gov/nuccore/93059904), which is involved in the stress response (Mochida et al., 2006), suggesting the important role this locus may play in response to metribuzin stress.

In comparison to the study done by Bhoite et al. (2018), who reported metribuzin tolerance QTL on chromosomes 1A, 2D, and 4A, we detected six new loci in this study. Blasting the QTL flanking markers with the wheat reference genome RefV1.0 revealed that the 2D QTL overlapped in the two studies, as a flanking marker “DArT1219191” of *QMCC.UWA.2DS* fell into the interval of *Qsns.uwa.2DS*. However, no candidate genes were suggested for this QTL as the physical position of “DArT3064546”, another flanking marker of *QMCC.UWA.2DS*, was too far away, making the physical interval of the QTL too large (617,964,302 bp) for identifying any possible candidate genes. We suggest that the difference between the Chinese Spring (reference genome) and the parental cultivars used in this mapping study might be due to chromosomal fragment translocations. Although both studies reported QTL on chromosome 4A, their physical positions were far from each other, suggesting that chromosome 4A might harbor many genes responsible for metribuzin tolerance. Bhoite et al. (2019) later reported putative genes for metribuzin tolerance on chromosomes 2A, 2D, 3B, 4A, 4B, 7A, 7B, and 7D. We also identified QTL with candidate genes on chromosomes 2A, 3B, and 4A, but at different physical positions. Shi et al. (2020) investigated genomic regions underlying the tolerance to two other herbicides (carfentrazone-ethyl and tribenuron-methyl) and detected QTL on chromosomes 1B, 2D, 5B, 5D, 6D, and 7D in wheat. No overlapping was found with the QTL identified in this study, suggesting different regulatory mechanisms for the tolerance to different herbicides.

Controlling weeds effectively is vital for wheat production, especially in areas with a Mediterranean climate, such as Australia. Herbicides are the main management method for controlling weeds in crops. Thus, understanding the mechanisms of herbicide tolerance and breeding varieties with high herbicide resistance is critical for future economic food production. This study revealed that chlorophyll traits can be used to select for herbicide tolerance in wheat. The identified SNP markers and candidate genes will be useful for marker-assisted selection of metribuzin tolerance, which could be an efficient way to control weeds and further improve wheat production.

## Conclusion

A high-density genetic linkage map was constructed using 2,129 DArTseq markers. ICIM identified seven QTL, one each on chromosomes 2A, 2D, 3A, 3B, 4A, 5A, and 6A. One major QTL for metribuzin tolerance on chromosome 5A was further validated using KASP assays in the F_3_ validation population developed from a Chuanmai 25 × Dagger cross. Three major QTL (*Qrcc.uwa.2AS*, *Qrcc.uwa.5AL*, and *Qrcc.uwa.6AL*) explained a total of 33.90% of the phenotypic variation, and the identified candidate genes could facilitate marker-assisted selection of metribuzin tolerance in wheat. Moreover, the QTL could be fine-mapped to locate the causal genes responsible for metribuzin tolerance for sustainable wheat production.

## Data Availability Statement

QTL data is available in figshare data repository portal, with a doi: 10.6084/m9.figshare.12570113.

## Author Contributions

LX and HL contributed equally to this work. LX, HL, PS, and GY designed and conceived the study. LX, HL, GL, JW, and RB performed the experiments. LX, HL, and AK analyzed the data. LX, HL, and WZ wrote the manuscript.

## Funding

This work was supported by the Global Innovation Linkage program (GIL53853) from the Australian Department of Industry, Innovation and Science, and the China Scholarship Council (201808330058).

## Conflict of Interest

Author AK was employed by company Diversity Arrays Technology Pty. Ltd.

The authors declare that the research was conducted in the absence of any commercial or financial relationships that could be construed as a potential conflict of interest.

The reviewer HA declared a past co-authorship with several of the authors HL and GY to the handling editor.
