# Supplementary material for: QTL Mapping Using a High-Density Genetic Map to Identify Candidate Genes Associated With Metribuzin Tolerance in Hexaploid Wheat (Triticum aestivum L.)
Source: Front Plant Sci. 2020 Sep 17;11:573439. doi: 10.3389/fpls.2020.573439 (PMC7527527; doi:10.3389/fpls.2020.573439)
Supplement: Supplementary file 1 [file Image_1.pdf]

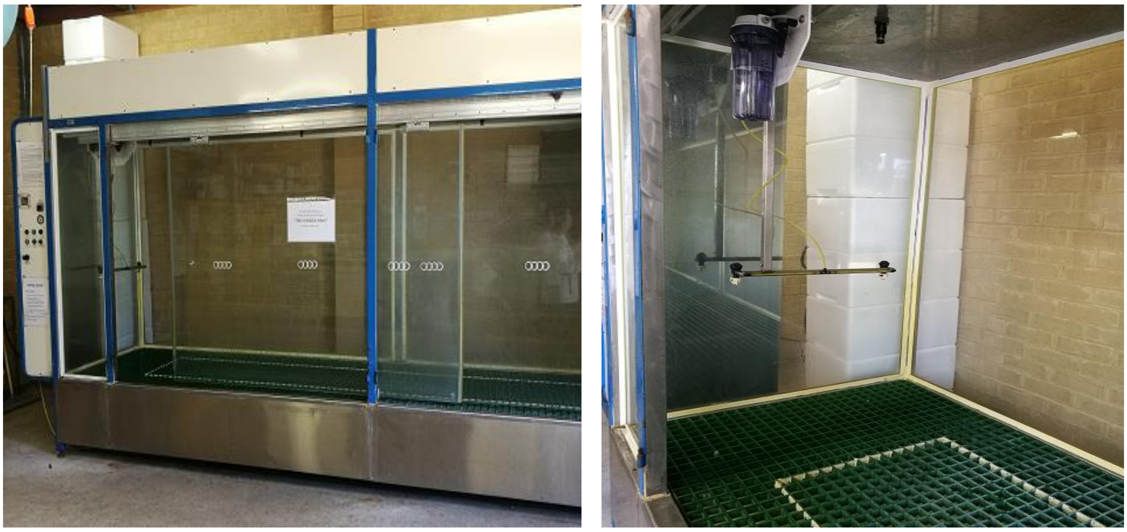

**FIGURE S1** The cabinet spray chamber (TeeJet XR11001 flat fan, Spraying Systems Co, Wheaton, IL, USA) used for metribuzin spray.
